# Identification of Potent Chloride Intracellular Channel Protein 1 Inhibitors from Traditional Chinese Medicine through Structure-Based Virtual Screening and Molecular Dynamics Analysis

**DOI:** 10.1155/2017/4751780

**Published:** 2017-09-25

**Authors:** Wei Wang, Minghui Wan, Dongjiang Liao, Guilin Peng, Xin Xu, Weiqiang Yin, Guixin Guo, Funeng Jiang, Weide Zhong, Jianxing He

**Affiliations:** ^1^Department of Thoracic Surgery, First Affiliated Hospital of Guangzhou Medical University, Guangzhou 510120, China; ^2^Guangzhou Institute of Respiratory Diseases and China State Key Laboratory of Respiratory Disease, Guangzhou 510120, China; ^3^National Respiratory Disease Clinical Research Center, Guangzhou 510000, China; ^4^Southern Medical University, Guangzhou 510000, China; ^5^Department of Radiation Oncology, First Affiliated Hospital of Guangzhou Medical University, Guangzhou 510120, China; ^6^National Supercomputer Center in Guangzhou, Guangzhou 510000, China; ^7^Department of Urology, Guangdong Key Laboratory of Clinical Molecular Medicine and Diagnostics, Guangzhou First People's Hospital, Guangzhou Medical University, Guangzhou 510180, China

## Abstract

Chloride intracellular channel 1 (CLIC1) is involved in the development of most aggressive human tumors, including gastric, colon, lung, liver, and glioblastoma cancers. It has become an attractive new therapeutic target for several types of cancer. In this work, we aim to identify natural products as potent CLIC1 inhibitors from Traditional Chinese Medicine (TCM) database using structure-based virtual screening and molecular dynamics (MD) simulation. First, structure-based docking was employed to screen the refined TCM database and the top 500 TCM compounds were obtained and reranked by *X*-Score. Then, 30 potent hits were achieved from the top 500 TCM compounds using cluster and ligand-protein interaction analysis. Finally, MD simulation was employed to validate the stability of interactions between each hit and CLIC1 protein from docking simulation, and Molecular Mechanics/Generalized Born Surface Area (MM-GBSA) analysis was used to refine the virtual hits. Six TCM compounds with top MM-GBSA scores and ideal-binding models were confirmed as the final hits. Our study provides information about the interaction between TCM compounds and CLIC1 protein, which may be helpful for further experimental investigations. In addition, the top 6 natural products structural scaffolds could serve as building blocks in designing drug-like molecules for CLIC1 inhibition.

## 1. Introduction

Chloride intracellular channel 1 (CLIC1), a newly discovered member of the highly evolutionarily conserved CLIC family of chloride ion channel proteins, was first cloned because of its increased expression in activated macrophages [1]. CLIC1 located within the plasma membrane and other internal cell membranes are involved in diverse physiological processes [2, 3]. The CLIC family have seven members: CLIC1 (NCC27), CLIC2, CLIC3, CLIC4, and CLIC5A whose sequences are highly conserved across species and two larger variants, CLIC5B and CLIC6 [4, 5]. They are known to participate in many physiological processes, the control of absorption and secretion of salt, acidification of organelles, and the regulation of cell volume and membrane potentials [6]. Malfunction of any of these channel proteins can lead to severe disease states [7].

Previous studies suggested CLIC1 appears to have a broad tissue distribution; it has been most intensely studied in various tumor tissues like gastric cancer [8], colon cancer [9], lung cancer [10], liver cancers [11], and glioblastoma. All of these studies showed that the high expression of CLIC1 has important association with tumor invasion, metastasis, and prognosis. For example, CLIC1 is involved in the development of most aggressive human tumors, including glioblastoma (GBM) [12] and lung adenocarcinoma [10]. CLIC1 is mainly localized in cytosol in resting cells, while it is progressively oxidized and recruits transiently to the plasma membrane, where it functions as a chloride selective ion channel, during cell cycle progression [13]. In vivo and in vitro proliferation of GBM cancer stem cells depends on CLIC1 activity, and its inhibition reduces tumor development in animal models.

Recently, Gritti et al. [14] discovered that CLIC1 is a direct target of metformin in human GBM cells. They identified that CLIC1 is not only a modulator of cell cycle progression in human GBM stem cells but also the main target of metformin's antiproliferative activity, paving the way for novel and necessary pharmacological approaches to GBM treatment. Therefore, CLIC1 is a potential prognostic marker and drug therapy target for diverse malignant tumors.

Although CLIC1 represents an emerging therapeutic target and shows important significance in clinical diagnosis, only few CLIC1 inhibitors have been reported to date [6, 15]. The most recognized CLIC1 inhibitor is IAA-94, known as a glutathione transferase (GST) binding molecule [16], and based on ethacrynic acid. Similarly, it is not entirely surprising that the CLICs are members of the GST superfamily. As well as predictions based on sequence similarity, p64, the first identified CLIC, was purified and characterized by its ability to bind the chloride channel inhibitor. In fact, the affinity purification experiments first isolated p64 and GST concurrently.

Herein, we focus on identification of potent CLIC1 inhibitors. To achieve this goal, the integrated in silico protocol, including docking-based high throughput screening, MD simulations, and Molecular Mechanics/Generalized Born Surface Area (MM-GBSA) analysis, was designed to discover new potent CLIC1 inhibitors from the world's largest TCM database. Based on our strategy, six TCM compounds were predicted as promising CLIC1 inhibitors, which may become the new lead compounds or drug candidates in the future.

## 2. Materials and Methods

### 2.1. TCM Database and Receptor Preparation

A total of 57423 ligand molecules were obtained from the TCM database @Taiwan (http://tcm.cmu.edu.tw) [17–19] and refined as the following protocol. First, we did the pretreatment for each molecular structure, including removing the counterions, solvent moieties and salts, adding hydrogen atoms, and optimizing the structures based on the MMFF94 force field using MOE (version 2010.10, Chemical Computing Group, Inc., Canada) [20–23]. Second, the refined database was filtered using drug-like analysis including Lipinski rules of five and PAINS assay http://cbligand.org/PAINS) [24, 25]. Then, all molecules were automatically converted to PDBQT format. Open Babel software (http://openbabel.org/wiki/) and in-house python script were used for manipulating the various chemical formats of ligand molecules [26].

For the docking simulations, the cocrystallized structure of human CLIC1 with glutathione (PDB code: 1K0N) [7] with a resolution factor of 1.80 Å was retrieved from RCSB protein data bank and prepared in three steps [7]. First, all native ligands, ions, and crystalline water were removed from the cocrystallized structure of CLIC1. Second, the missing hydrogen atoms were added [27]. Finally, the protein file was automatically prepared in PDBQT format. Molecular Graphics Laboratory (MGL Tool) software was applied in preparing all of the structure parameters of CLIC1 protein.

### 2.2. Docking-Based Virtual Screening

AutoDock Vina was employed to screen the refined 9033 TCM library against CLIC1 [28]. The docking site was defined on glutathione active binding site (−1.343, −6.385, 32.927 Å) and the grid box was set as 25 × 25 × 25 Å in *x*, *y*, and *z* directions. During docking process, the semiflexible docking simulations were performed employing Lamarckian genetic algorithm, and the receptor was kept rigid, while the ligands were flexible to rotate and explore the most probable binding conformations. After docking-based virtual screening, the top 5000 TCM compounds with docking score were obtained [29, 30].

The top 5,000 TCM compounds were resorted by *X*-Score. Again, the top 500 compounds were selected from the 5,000 reranked compounds for clustering. Clustering and visual analyses were carried out to remove redundancies resulting from similar structures and check the docking poses and interactions between the ligands and CLIC1 [20]. Finally, 30 compounds were selected for further analysis.

### 2.3. Molecular Dynamics Simulation

Molecular docking method only reflects a possibly instantaneous binding mode which may not be reasonable/stable between a ligand and a receptor. Therefore, MD simulations were applied to perform further evaluation of the binding stabilities between all the top 30 TCM and their receptor CLIC1. MD simulations were performed to investigate the binding patterns of the virtual screening top 30 hits using the PMEMD module in AMBER 12 software accelerated by running on a GPU system, the NVIDIA CUDA processor [31]. The CLIC1 protein complexes with docked structures of top hits were used as the initial coordinates for MD simulations. The protein and top hits were applied with ff99SB and Generalized Amber Force Field (GAFF), respectively. The partial charges of ligands were computed using the HF/6-31 G^*∗*^ basis set from GAUSSIAN09 and refined by RESP calculation using the antechamber module of the AMBER 12 package [20]. Each system was solvated in a truncated octahedron box of TIP3P water molecules with a margin distance of 10 Å. Periodic boundary conditions were applied. Neutralizing counterions were added to the simulation system.

To remove possible steric stresses, each system was minimized for 2,000 steps with the steepest descent method, followed by application of conjugate gradients for another 2,000 steps. Each system was linearly heated from 0 to 310 K using a Langevin thermostat, with a collision frequency of 5.0 ps^−1^ and harmonic restraints of 4 kcal/mol/Å2 on the backbone atoms over 50 ps and then equilibrated for 50 ps at 310 K using the NVT ensemble. A production simulation run for 5 ns was performed using the NPT ensemble. Coordinate trajectories were saved every 1 ps for the whole MD runs. The temperature was kept at 310 K by means of a weak coupling algorithm [23]. Covalent bonds involving hydrogen were constrained using the SHAKE algorithm.

### 2.4. Binding Free Energy Analysis

To provide insight into the interaction energies and energetic stabilities of the CLIC1 and TCM compounds, the MM/GBSA method [32] in the AMBER 12 was used to calculate the binding free energies for 30 hits. Detailed calculations and analyses can be found in the previous studies [33–36]. The final top 6 hits were selected as potent CLIC1 inhibitor according to the ranked binding free energy results.

## 3. Results and Discussion

### 3.1. Binding Domain Analysis

**Figure 1:**
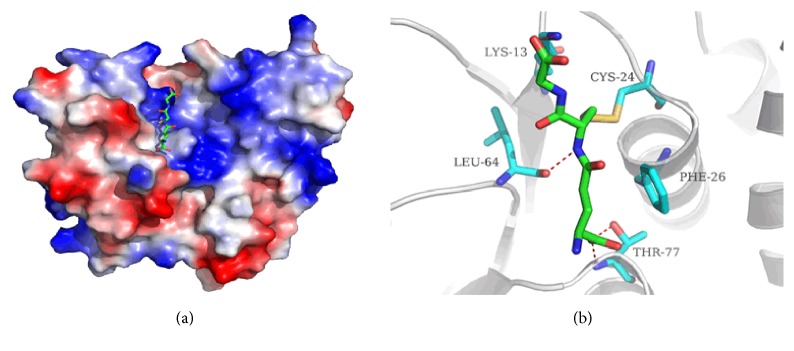
Structure of the glutathione_CLIC1 complex. (a) shows the electrostatic potential on the molecular surface of glutathione-bound CLIC1. (b) shows the interactions between the glutathione and the sounding residues.

The electrostatic potential representation structure of glutathione-CLIC1 complex is shown in Figure 1(a). The green molecule is glutathione (GSH) surrounded by the basic lobes of the N and C domains at the edge of a slot at the top of the molecule (Figure 1(a)). According to the previous study [7], the N-domain of CLIC1 has a well-conserved glutaredoxin-like site for covalently interacting with GSH. The thiol of Cys24 in CLIC1 is likely to be a highly reactive thiolate with a low pKa due to its position at the amino terminus of helix h1 (Figure 1(b)) [37].

**Figure 2:**
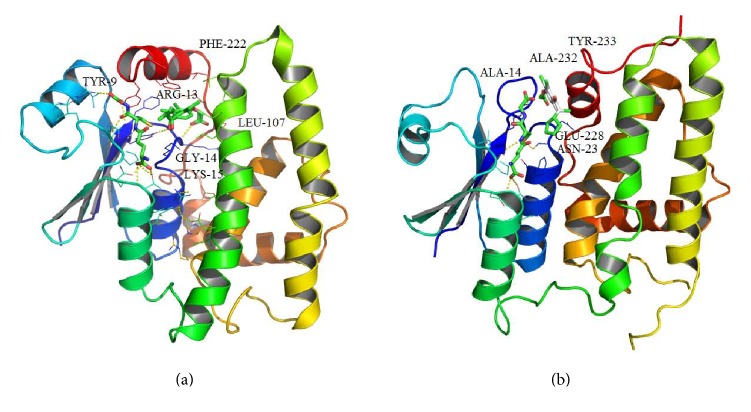
Receptor-ligand interactions of compound. (a) Glutathione transferase A1-1 complexed with glutathione (left) ethacrynic acid (right) conjugate (PDB code: 1GSE). (b) Chloride intracellular channel 1 (CLIC1) complexed with glutathione (left) IAA-94 (right) docking result (PDB code: 1K0N).

The interactions between GST and ethacrynic acid inhibitor compared with CLIC1 and IAA-94 inhibitor were shown in Figure 2[16]. The structure of the soluble form of CLIC1 indicates that it belongs to the GST superfamily [7]. Hence, the mechanisms of IAA-94, a well-characterized CLIC1 inhibitor, and GSH in CLIC1 are likely to be related in ethacrynic acid and GSH in GST [7, 38]. Ethacrynic acid binds to GST at the electrophilic substrate site (“H-site”), surrounded by TYR-9, ARG-13, GLY-14, LYS-15, LEU-107, and PHE-222, which is adjacent to the GSH binding site (Figure 2(a)) [39]. In GSTs, the H-site is formed by the loop connecting *β*-strand s1 to helix h1 and helix h4 plus the carboxyl terminus (the “walls”) and helix h9 (the “lid”). This corresponds to the more open and elongated slot in CLIC1 (Figures 1(a) and 2(b)). Due to its structural homology to ethacrynic acid, IAA-94 is bound to CLIC1 protein in the slot adjacent to the GSH binding site, surrounded by ALA-14, ASN-23, GLU-228, ALA-232, and TYR-233 [40]. To ensure docking reliability, the validation of docking performed based on the available crystal structure. GSH was docked to the apo-protein and the RMSD value of XRD and GSH is 1.351, suggesting the docking method used in the present study is reliable. Therefore, the grid center was defined by glutathione active binding domain and grid box was set as 25 × 25 × 25 points in *x*, *y*, and *z* directions, which contains the slot of binding site of CLIC1 potential inhibitors.

### 3.2. Virtual Screening Result

**Figure 3:**
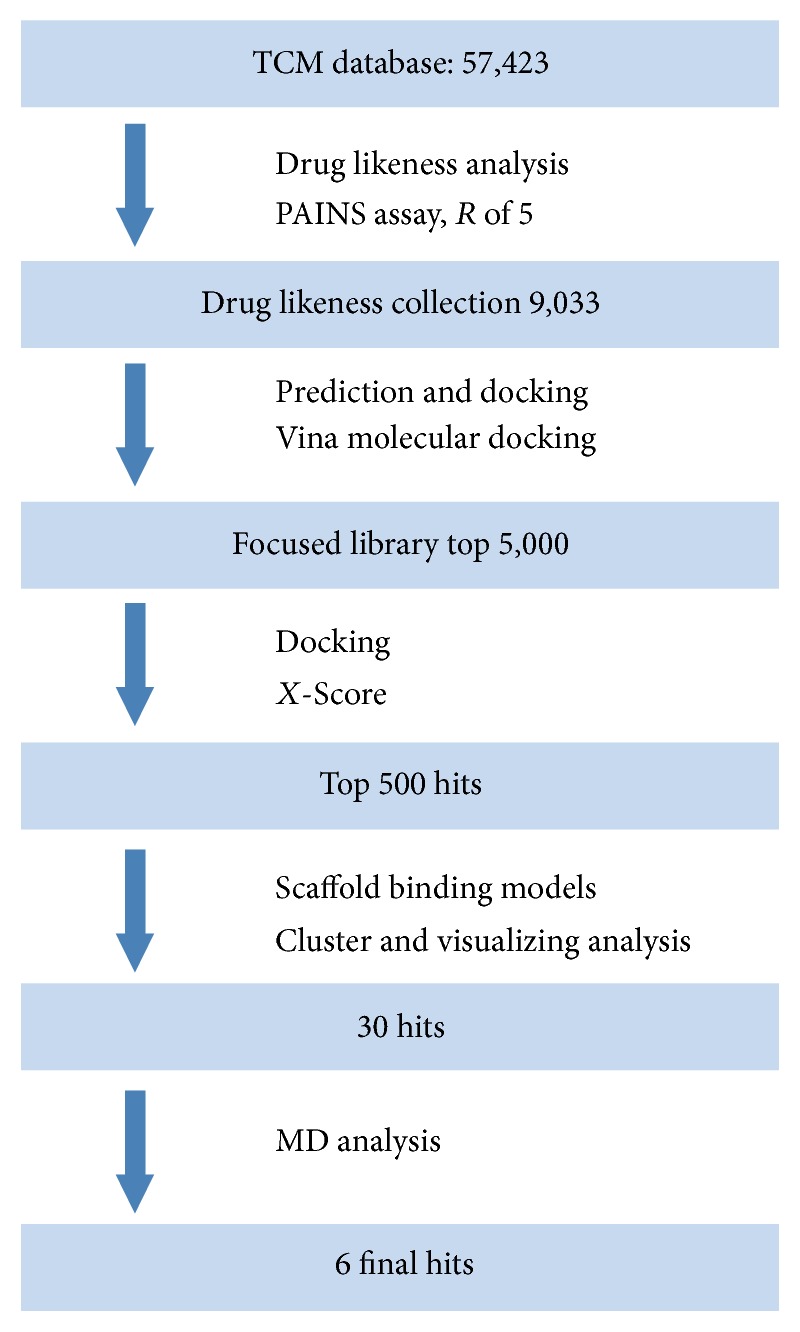
Protocol flowchart of CLIC1 inhibitors discovery strategy in this study.

Virtual screening is gaining increasingly important influence in modern drug discovery. It can be used to screen large compound databases and reduce large numbers of compounds to smaller subsets that are more likely to contain biologically active compounds. In this work, we designed a systematic strategy for identifying natural products CLIC1 inhibitors using structure-based VS and MD simulation. The detailed flowchart is shown in Figure 3. Among the MOL2 files in TCM database, 9,033 natural products were obtained from the mother TCM database containing 57,423 using the Lipinski rules and PAINS assay filtering. The Lipinski rule states that “drug-like” molecules must satisfy the conditions below at the same time: log⁡*P* ≤ 5, 150 ≤ molecular weight ≤ 500, number of hydrogen bond donors ≤ 5, number of hydrogen bond acceptors ≤ 10, and number of rotatable bonds ≤ 10 [41]. Also, PAINS-Remover is used to remove the Pan Assay Interference Compounds (PAINS) from screening libraries and for their exclusion in bioassays. This server will facilitate data-sharing and information exchange among UPCMLD scientific research communities with online structure search functions and data analysis tools implemented for removal of PAINS [25]. Then, the 9033 TCM compounds were docked into the binding site of CLIC1 by AutoDock Vina, followed by ranking according to their binding energy. The top 5000 molecules were selected for further* X*-Score analysis (Figure 3).

*X*-Score software computes a binding score for a given protein-ligand complex structure, and this binding score correlates with experimental binding constants well. Three individual empirical scoring functions have been implemented in* X*-Score software, namely, HPScore, HMScore, and HSScore [29]. According to the results ranked by* X*-Score, the top 500 hits were kept. Then, the 500 TCM with top* X*-Scores were stored separately for clustering and visual analyses. These compounds were inspected to check whether they had interactions with the GSH binding pocket of CLIC1 protein. This step makes sure that selected candidates have not only a higher docking score but also a rational binding mode. An ECFP_4 fingerprint based clustering algorithm implemented in discovery studio 3.5 software (Accelrys, Inc., San Diego, CA) was applied for structure diversity analysis to ensure that the hits selected from the virtual screening were unique and unrepeated. Finally, 30 compounds were chosen for further MD analysis and their corresponding zinc codes were listed in Table 1.

### 3.3. Molecular Dynamics Results

**Figure 4:**
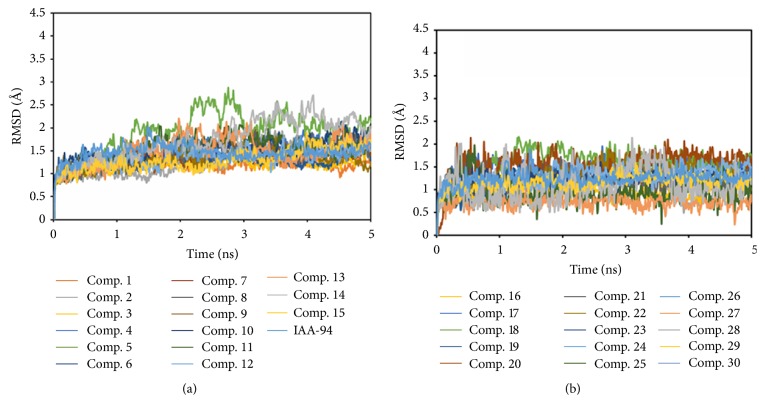
The RMSD (Å) trajectories of IAA-94 and top 30 TCM compounds in CLIC1 complexes during 5 ns MD simulation.

**Figure 5:**
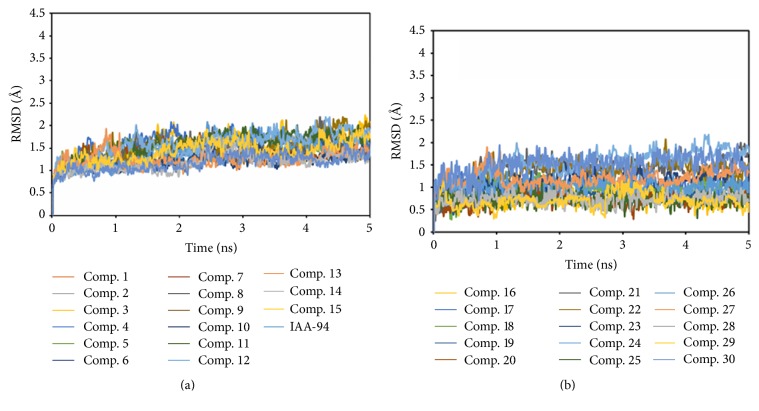
The RMSD (Å) trajectories of IAA-94 and top 30 TCM compounds in binding site (residues within 6.5 Å to the ligands) of CLIC1 complexes during 5 ns MD simulation.

The RMSD values of the complexes of the 30 hits and CLIC1 during the MD simulations were monitored in Figure 4. Meanwhile, the RMSD values of the known inhibitor IAA-94 and CLIC1 complex during the MD simulations were also drawn in Figure 4(a). In order to provide the explanation, 5 ns of MD simulations is really capable of representing the equilibrated ligand-protein-complex. The RMSD (Å) trajectories of IAA-94 and top 30 TCM compounds in binding site (residues within 6.5 Å to the ligands) of CLIC1 complexes during 5 ns MD simulation were drawn in Figure 5. From Figure 5, we can see all the RMSD curves in the selected part (residues within 6.5 Å to the ligands) were stable after 1 ns. Here, we employed the MM-GBSA method encoded in Amber 12 to calculate the ligand binding free energies and rescore the docking hits. The detailed MM-GBSA scores results are listed in Table 1 [42]. As shown in Table 1, we can clearly achieve the fact that the top 6 TCM hits have relative bigger binding free energy, indicating they are potential CLIC1 inhibitors.

The detailed binding energy profiles of three TCM hits (16, 22, and 20) are shown in Table 2. Both van der Waals and electrostatic components play key roles in 16 binding, and the van der Waals contribution (−25.28 kcal/mol) is equal to the electrostatic component (−24.52 kcal/mol). Electrostatic solvation (Δ*G*ele, solv) disfavors binding because of the desolvation penalty for 16 and CLIC1. The nonpolar component of solvation (Δ*G*nonpol, solv), which corresponds to the burial of solvent-accessible surface area (SASA) upon binding, provides a slightly favorable contribution. For compound 22, nonbonded electrostatics component is approximately 5-fold greater that the van der Waals component, while the van der Waals component is approximately 22-fold greater the electrostatics component in 16-CLIC1 complex. Electrostatic solvation (Δ*G*ele, solv) disfavors binding for both 20, 22, and CLIC1.

The binding free energy of positive control IAA-94 and CLIC1 complex were calculated using the same method MM/GBSA (Table 2). Here, we can conclude the van der Waals contribution (−18.47 kcal/mol) of compound 22 is approximately equal to van der Waals contribution (−18.83 kcal/mol) of IAA-94. Also, the van der Waals contributions (−25.28 kcal/mol) of compound 16 and compound 20 (−28.67 kcal/mol) were greater than our positive control. In conclusion, the binding energy of top 3 hits is greater than the known inhibitor.

### 3.4. Binding Mode Analysis for Six Potential Candidates

**Figure 6:**
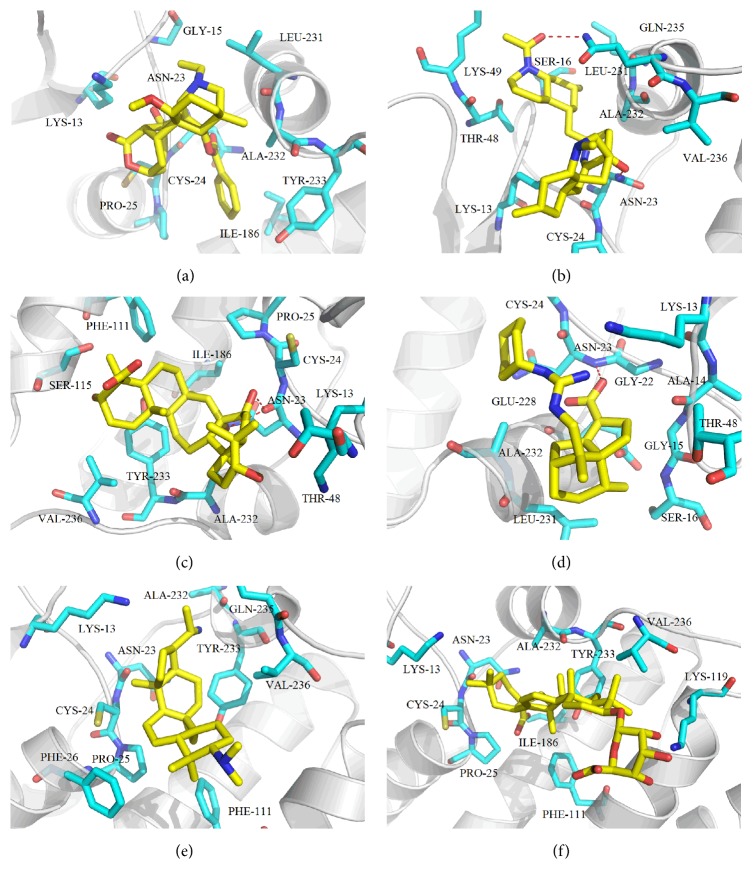
Predicting binding modes for top 6 TCM compounds 16 (a), 22 (b), 20 (c), 14 (d), 2 (e), and 24 (f). Hydrogen bonds are depicted by red dotted lines. Top hits compounds are the yellow molecules.

**Figure 7:**
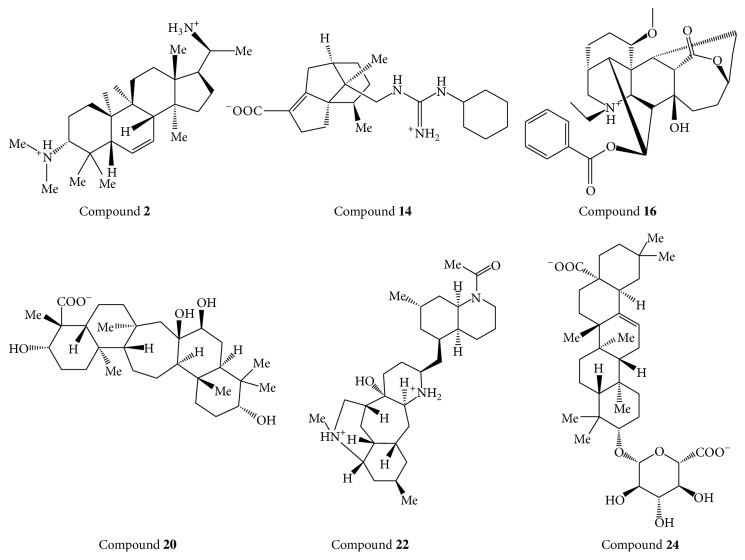
The molecular structure of the top 6 TCM compounds.

The predicting binding modes for the top 6 TCM compounds were illustrated in Figure 6. And the molecular structures of the top 6 TCM compounds were shown in Figure 7. As shown in the Figure 6, compound 16 is surrounded by hydrophobic amino acids including CYS-24, ILE-176, LEU-221, ALA-222, and TYR-223. Compound 22 can form two hydrogen bonds with ASN-23 and GLU-225 (Figure 6(b)), respectively. It also was surrounded by hydrophobic amino acids (CYS-24, LEU-221, and VAL-226). Meanwhile, compounds 20 and 14 can also form hydrogen bond with the residue ASN-23 (Figures 6(c) and 6(d)). They were in a hydrophobic chamber surrounded by SER-16, GLY-22, CYS-24, THR-48, PHE-111, ILE-176, LEU-221, ALA-222, TYR-223, and VAL-226. The van der Waals interaction is major contribution for compound 2 and 24 binding with hydrophobic amino acids of CLIC1 (CYS-24, PHE-26, PHE-111, ALA-222, TYR-223, and VAL-226) (Figures 6(e) and 6(f)). To analyze the six ligand-protein binding modes in Figure 6, we can conclude that hydrogen bonds and van der Waals play a key role in protein and small molecules interaction. All of these binding modes analysis results are consistent with the binding energy results. Hence, we propose the six TCM compounds (Figure 7) as potential candidates for further study in drug development process with the CLIC1 protein against several types of cancer [43].

## 4. Conclusion

This study aims to investigate the potent lead TCM candidates for CLIC1 protein inhibitors against cancer. We introduced a systematic structure-based VS and MD analysis study about potential inhibitors against CLIC1 from the world's largest natural products TCM database. 30 TCM hits were selected through molecular docking-based virtual screening,* X*-Score, cluster, and visualizing analysis. 6 of 30 TCM hits are refined through 5 ns MD simulations and MMGB-SA binding energy analysis. The detailed binding modes of 6 TCM candidates were illustrated and discussed. We hope our results may inspire medicinal chemists to further develop these potential CLIC1 inhibitors into lead compounds for the treatment of solid cancers in the near future.

## Figures and Tables

**Table 1:** 30 TCM compounds ranked by their binding free energies.

Comp.	Zinc code	Δ*G*_bind_ (Kcal/mol)
1	ZINC85569445	−12.55
2^*∗*^	ZINC95909928	−47.36
3	ZINC95909488	−7.37
4	ZINC70455535	−20.23
5	ZINC15211150	−1.19
6	ZINC44406126	−9.90
7	ZINC85549771	−21.88
8	ZINC85492224	−26.92
9	ZINC13490979	−9.19
10	ZINC95909715	−11.58
11	ZINC85532205	−19.46
12	ZINC85569698	−6.76
13	ZINC95919401	−44.90
14^*∗*^	ZINC85549124	−49.46
15	ZINC14652472	−7.87
16^*∗*^	ZINC95909751	−84.53
17	ZINC70455083	−4.85
18	ZINC33833039	−35.68
19	ZINC95919003	−6.71
20^*∗*^	ZINC44351718	−64.41
21	ZINC04071656	−10.35
22^*∗*^	ZINC95910575	−73.10
23	ZINC70451186	−4.73
24^*∗*^	ZINC49832948	−45.14
25	ZINC95910338	−13.71
26	ZINC95909921	−17.27
27	ZINC85543198	−23.60
28	ZINC33832995	−14.33
29	ZINC05765515	−7.06
30	ZINC42965023	−19.25

Δ*G*_bind_: final estimated binding free energy based on MM-GBSA calculations. *∗* indicated the top 6 compounds ranked by binding free energy.

**Table 2:** The detailed binding free energy for IAA-94 and top 3 *TCM* compounds based on MM-GBSA method.

Energy terms	Binding free energy (Kcal/mol) (SEM)			
	IAA-94	Compound **16**	Compound **22**	Compound **20**
Δ*E*_vdw_^a^	−18.83 (0.11)	−25.28 (0.25)	−18.47 (0.30)	−28.67 (0.17)
Δ*E*_ele_^b^	−32.80 (0.65)	−24.52 (1.22)	−91.54 (0.52)	−1.29 (0.68)
Δ*E*_pol,solv_^c^	44.62 (0.61)	33.33 (1.24)	40.98 (0.41)	23.05 (0.56)
Δ*E*_nonpol,solv_^d^	−2.09 (0.01)	−3.45 (0.03)	−4.07 (0.02)	−3.62 (0.02)
Δ*G*_gas_^e^	−51.64 (0.64)	−114.42 (1.42)	−110.00 (0.44)	−83.84 (0.74)
Δ*G*_solv_^f^	42.53 (0.61)	29.89 (1.22)	36.91 (0.40)	19.43 (0.56)
Δ*G*_bind_^g^	−9.10 (0.11)	−84.53 (0.35)	−73.10 (0.26)	−64.41 (0.38)

^a^Nonbonded van der Waals. ^b^Nonbonded electrostatics. ^c^Polar component to solvation. ^d^Nonpolar component to solvation. ^e^Total gas phase energy. ^f^Sum of nonpolar and polar contributions to solvation. ^g^Final estimated binding free energy calculated from the terms above. Standard errors of the mean are given in parentheses.

## References

[1] Valenzuela S. M., Martin D. K., Por S. B. (1997). Molecular cloning and expression of a chloride ion channel of cell nuclei. *Journal of Biological Chemistry*.

[2] Al-Awqati Q. (1995). Chloride channels of intracellular organelles. *Current Opinion in Cell Biology*.

[3] Jentsch J T., Günther W. (1997). Chloride channels: an emerging molecular picture. *Bioessays News & Reviews in Molecular Cellular & Developmental Biology*.

[4] Fanucchi S., Adamson R. J., Dirr H. W. (2008). Formation of an unfolding intermediate state of soluble chloride intracellular channel protein CLIC1 at acidic pH. *Biochemistry*.

[5] Tulk B. M., Kapadia S., Edwards J. C. (2002). CLIC1 inserts from the aqueous phase into phospholipid membranes, where it functions as an anion channel. *American Journal of Physiology - Cell Physiology*.

[6] Shi Z.-H., Zhao C., Wu H., Wang W., Liu X.-M. (2011). CLIC1 Protein a candidate prognostic biomarker for malignant-transformed hydatidiform moles. *International Journal of Gynecological Cancer*.

[7] Harrop S. J., DeMaere M. Z., Fairlie W. D. (2001). Crystal structure of a soluble form of the intracellular chloride ion channel CLIC1 (NCC27) at 1.4 Å resolution. *Journal of Biological Chemistry*.

[8] Chen C. D., Wang C. S., Huang Y. H. (2007). Overexpression of CLIC1 in human gastric carcinoma and its clinicopathological significance. *Proteomics*.

[9] Petrova D. T., Asif A. R., Armstrong V. W. (2008). Expression of chloride intracellular channel protein 1 (CLIC1) and tumor protein D52 (TPD52) as potential biomarkers for colorectal cancer. *Clinical Biochemistry*.

[10] Wang W., Xu X., Wang W. (2011). The expression and clinical significance of CLIC1 and HSP27 in lung adenocarcinoma. *Tumor Biology*.

[11] Huang J.-S., Chao C.-C., Su T.-L. (2004). Diverse cellular transformation capability of overexpressed genes in human hepatocellular carcinoma. *Biochemical and Biophysical Research Communications*.

[12] Setti M., Savalli N., Osti D. (2013). Functional role of CLIC1 ion channel in glioblastoma-derived stem/progenitor cells. *Journal of the National Cancer Institute*.

[13] Littler D. R., Harrop S. J., Fairlie W. D., Brown L. J., et al (2004). The intracellular chloride ion channel protein CLIC1 undergoes a redox-controlled structural transition. *Journal of Biological Chemistry*.

[14] Gritti M., Würth R., Angelini M. (2014). Metformin repositioning as antitumoral agent: selective antiproliferative effects in human glioblastoma stem cells, via inhibition of CLIC1-mediated ion current. *Oncotarget*.

[15] Goodchild S. C., Angstmann C. N., Breit S. N., Curmi P. M. G., Brown L. J. (2011). Transmembrane extension and oligomerization of the CLIC1 chloride intracellular channel protein upon membrane interaction. *Biochemistry*.

[16] Redhead C. R., Edelman A. E., Brown D., Landry D. W., Al-Awqati Q. (1992). A ubiquitous 64-kDa protein is a component of a chloride channel of plasma and intracellular membranes. *Proceedings of the National Academy of Sciences of the United States of America*.

[17] Kakarala K. K., Jamil K. (2015). Screening of phytochemicals against protease activated receptor 1 (PAR1), a promising target for cancer. *Journal of Receptors and Signal Transduction*.

[18] Brožič P., Turk S., Lanišnik Rižner T., Gobec S. (2009). Discovery of new inhibitors of aldo-keto reductase 1C1 by structure-based virtual screening. *Molecular and Cellular Endocrinology*.

[19] Chen C. Y. (2011). TCM Database@Taiwan: the world's largest traditional Chinese medicine database for drug screening *in silico*. *PLoS ONE*.

[20] Wang L., Chen L., Yu M. (2016). Discovering new mTOR inhibitors for cancer treatment through virtual screening methods and in vitro assays. *Scientific Reports*.

[21] Wang L., Li Y., Xu M. (2016). Chemical fragment-based CDK4/6 inhibitors prediction and web server. *RSC Advances*.

[22] Wang L., Le X., Li L. (2014). Discovering new agents active against methicillin-resistant Staphylococcus aureus with ligand-based approaches. *Journal of Chemical Information and Modeling*.

[23] Wang L., Gu Q., Zheng X., Ye J., Liu Z. (2013). Discovery of new selective human aldose reductase inhibitors through virtual screening multiple binding pocket conformations. *Journal of Chemical Information Modeling*.

[24] Lipinski C. A. (2004). Lead- and drug-like compounds: the rule-of-five revolution. *Drug Discovery Today: Technologies*.

[25] Baell J. B., Holloway G. A. (2010). New substructure filters for removal of pan assay interference compounds (PAINS) from screening libraries and for their exclusion in bioassays. *Journal of Medicinal Chemistry*.

[26] Zhao H., Gartenmann L., Dong J., Spiliotopoulos D., Caflisch A. (2014). Discovery of BRD4 bromodomain inhibitors by fragment-based high-throughput docking. *Bioorganic and Medicinal Chemistry Letters*.

[27] Maltarollo V. G., Togashi M., Nascimento A. S., Honorio K. M. (2015). Structure-based virtual screening and discovery of new PPAR*δ*/*γ* dual agonist and PPAR*δ* and *γ* agonists. *PLoS ONE*.

[28] Trott O., Olson A. J. (2010). AutoDock Vina: improving the speed and accuracy of docking with a new scoring function, efficient optimization and multithreading. *Journal of Computational Chemistry*.

[29] Wang R., Lai L., Wang S. (2002). Further development and validation of empirical scoring functions for structure-based binding affinity prediction. *Journal of Computer-Aided Molecular Design*.

[30] Wang R., Lu Y., Wang S. (2003). Comparative evaluation of 11 scoring functions for molecular docking. *Journal of Medicinal Chemistry*.

[31] Pearlman D. A., Case D. A., Caldwell J. W. (1995). AMBER, a package of computer programs for applying molecular mechanics, normal mode analysis, molecular dynamics and free energy calculations to simulate the structural and energetic properties of molecules. *Computer Physics Communications*.

[32] Kollman P. A., Massova I., Reyes C. (2001). Calculating structures and free energies of complex molecules: combining molecular mechanics and continuum models. *Accounts of Chemical Research*.

[33] Hou T., Wang J., Li Y., Wang W. (2011). Assessing the performance of the MM/PBSA and MM/GBSA methods. 1. The accuracy of binding free energy calculations based on molecular dynamics simulations. *Journal of Chemical Information and Modeling*.

[34] Kollman P. A., Massova I., Reyes C., Kuhn B., Huo S. Calculating structures and free energies of complex molecules.

[35] Hou T., Wang J., Li Y., Wang W. (2011). Assessing the performance of the molecular mechanics/Poisson Boltzmann surface area and molecular mechanics/generalized Born surface area methods. II. the accuracy of ranking poses generated from docking. *Journal of Computational Chemistry*.

[36] Xu L., Sun H., Li Y., Wang J., Hou T. (2013). Assessing the performance of MM/PBSA and MM/GBSA methods. 3. the impact of force fields and ligand charge models. *Journal of Physical Chemistry B*.

[37] Kortemme T., Creighton T. E. (1995). Ionisation of cysteine residues at the termini of model *α*-helical peptides. Relevance to unusual thiol pKa values in proteins of the thioredoxin family. *Journal of Molecular Biology*.

[38] Cameron A. D., Sinning I., L'Hermite G. (1995). Structural analysis of human alpha-class glutathione transferase A1-1 in the apo-form and in complexes with ethacrynic acid and its glutathione conjugate. *Structure*.

[39] Armstrong R. N. (1997). Structure, catalytic mechanism, and evolution of the glutathione transferases. *Chemical Research in Toxicology*.

[40] Landry D. W., Reitman M., Cragoe E. J., Al-Awqati Q. (1987). Epithelial chloride channel. Development of inhibitory ligands. *Journal of General Physiology*.

[41] Paramashivam S. K., Elayaperumal K., Natarajan B. B., Ramamoorthy M., Balasubramanian S., Dhiraviam K. (2015). In silico pharmacokinetic and molecular docking studies of small molecules derived from *Indigofera aspalathoides* Vahl targeting receptor tyrosine kinases. *Bioinformation*.

[42] Chen L., Wang L., Gu Q., Xu J. (2014). An in silico protocol for identifying mTOR inhibitors from natural products. *Molecular Diversity*.

[43] Wang Wei Identification of Potent Chloride Intracellular Channel Protein 1 Inhibitors from Traditional Chinese Medicine through Structure-Based Virtual Screening and Molecular Dynamics Analysis.

